# Heterotopic Ossification: Clinical Features, Basic Researches, and Mechanical Stimulations

**DOI:** 10.3389/fcell.2022.770931

**Published:** 2022-01-25

**Authors:** Yili Xu, Mei Huang, Wenzhen He, Chen He, Kaixuan Chen, Jing Hou, Min Huang, Yurui Jiao, Ran Liu, Nanyu Zou, Ling Liu, Changjun Li

**Affiliations:** ^1^ Department of Endocrinology, Endocrinology Research Center, The Xiangya Hospital of Central South University, Changsha, China; ^2^ National Clinical Research Center for Geriatric Disorders (Xiangya Hospital), Changsha, China; ^3^ Key Laboratory of Organ Injury, Aging and Regenerative Medicine of Hunan Province, Changsha, China

**Keywords:** heterotopic ossification, mechanical loading, bone, stem cell fate, bone formation

## Abstract

Heterotopic ossification (HO) is defined as the occurrence of extraskeletal bone in soft tissue. Although this pathological osteogenesis process involves the participation of osteoblasts and osteoclasts during the formation of bone structures, it differs from normal physiological osteogenesis in many features. In this article, the primary characteristics of heterotopic ossification are reviewed from both clinical and basic research perspectives, with a special highlight on the influence of mechanics on heterotopic ossification, which serves an important role in the prophylaxis and treatment of HO.

## Introduction

Heterotopic ossification (HO) is a complicated pathologic process causing the formation of extra-skeletal bone in soft tissues, such as muscle, peri-articulations, ligaments, and tendons. It is commonly recognized as a complication after trauma, surgery, blast, spinal cord injury, and other stress damages (Shimono et al., 2011; Regard et al., 2013; Ranganathan et al., 2015; Wang et al., 2016). Heterotopic ossification was first labeled as “paraosteoarthropathy” by French physicians Dejerne and Ceillier, being a consequence of traumatic paraplegia of patients during World War I, and was further observed among soldiers returning from Iraq and Afghanistan (Naraghi et al., 1996; Forsberg et al., 2009; Potter et al., 2010; Forsberg et al., 2014). In severe cases, complete bony ankylosis as a result of HO is quite common, and more than 20% of patients appear overt dysfunction in soft-tissue, joint, or suffer from chronic pain; The HO morbidity of patients with traumatic brain injury almost reach 50% (Vanden Bossche and Vanderstraeten, 2005; Balboni et al., 2006; Zhang et al., 2014; Xu et al., 2018).

Inquiry about the underlying mechanism, such as cellular and mechanical processes, and earlier diagnoses as well as more effective treatments, is the hotspot of current research. Scientists analyze the proteomic biomarkers to identify early diagnostic indexes based on high-throughput mass spectrometry and antibody arrays; Doctors seek to develop efficacious prophylactic management and specific treatments via physical therapy, pharmaceutical intervention, operation, and radiation (Yuan et al., 2009; Coons and Godleski, 2013; Cheng et al., 2017; Gomez-Puerto et al., 2019; Botman et al., 2020). Moreover, patients with a high incidence of traumatic heterotopic ossification, such as fractures and hip joint arthroplasty, need to undergo prolonged postoperative immobilization or early rehabilitation exercises. Post-traumatic motion and mechanical loading are closely related to the occurrence of heterotopic ossification. The role of passive motion rehabilitative therapy after trauma, fracture, or invasive surgery for heterotopic ossification is still controversial. In this review, we elaborate on the clinical features and the fundamental biological mechanisms of HO, and for the first time summarize the separate influences of mechanical stimulations on HO based on up-to-date researches.

## Clinical Features of Heterotopic Ossification

### Epidemiology

HO is often divided into three categories: traumatic, neurogenic, and genetic. The prevalence of traumatic-induced HO following burn injury has been reported to range from 0.2 to 4%, and up to 90% following the total hip joint arthroplasty or acetabular fractures (Cipriano et al., 2009; Maender et al., 2010; Rath et al., 2013; Medina et al., 2014; Medina et al., 2015). The predilection age of trauma-induced HO is 20–40 years old. Approximately half of HO occurs at this age. However, the other half of the HO could present dispersedly from infancy to late adulthood (Ackerman, 1958; Elmas and Shrestha, 2017; Xu et al., 2017; Meyers et al., 2019; Kaliya-Perumal et al., 2020). The morbidity of heterotopic ossification following central neurologic injury has been calculated to range from 10 to 53% (Teasell et al., 2010). Most studies regard traumatic brain-injured patients and spinal cord injured patients as the same category. And the prevalence of genetic HO, including fibrodysplasia ossificans progressiva (FOP), progressive osseous heteroplasia (POH), and Albright’s hereditary osteodystrophy (AHO) (Shore and Kaplan, 2010), is extremely rare, affecting 1 in 2,000,000 people (Baujat et al., 2017). However, genetic HO is consensually regarded as the most severe HO disease in humans (Qi et al., 2017; Kaliya-Perumal et al., 2020). Male sex, the amount, and the type of motion could also raise the risk of HO. Men are slightly more vulnerable to HO with a sex ratio of 3:2 (Meyers et al., 2019), perhaps due to the various muscle mass, differential level of physical activity, repetitive mechanical stress working as “microtrauma”, and distinct hormonal signaling pathways affecting osteogenesis (Ranganathan et al., 2015; Ko et al., 2016; Malca et al., 2018; Dowdell et al., 2020; Rüdiger et al., 2020).

### Clinical Presentation

The typical clinical features of HO include the limited range of motion around the involved joint, complete bony ankylosis in severe cases, and deformity in the cervical spine, elbow, shoulder, fingers, jaw exostosis, or temporomandibular joint ankylosis (TMJA) (Zhao et al., 2020). HO could occur almost anywhere in the body, as long as it is associated with the periosteum. Typically, HO initiates away from the periosteum, and then fuse to the periosteum as a secondary feature (Meyers et al., 2019). But it is rare to observe HO in some anatomic tissues, such as the viscera or the diaphragm. This might be due to the lack of pluripotent stem cells in these sites or because these sites are not mechanically stimulated as often as the peri-articular areas prone to heterotopic ossification. Moreover, HO can only be detected as an asymptomatic finding on a radiograph. It is quite challenging to identify the potential biomarkers for early disease detection and monitoring, let alone the symptom present with complications that usually confound diagnosis (Crowgey et al., 2018). There are several ways to classify HO diseases. Four levels of classification for HO around the hip were set by Brooker to indicate the severity (Brooker et al., 1973). The Hastings and Graham classification system classifies HO at the elbow into three grades based on clinical and radiographic data (Hastings and Graham, 1994).

The presentations of genetic HO are more serious than traumatic-induced HO. Almost all FOP patients reported to date were caused by *Acvr1* mutation, and showing abnormality early. *Acvr1* gene locates on chromosome 2 (2q23-24) and encodes a bone morphogenetic protein (BMP) type 1 receptor, which is generally considered to be the major regulator in HO pathophysiology (Wang et al., 2016; Haupt et al., 2019; Meyers et al., 2019; Pearson et al., 2019; Stanley et al., 2019; Botman et al., 2020; Kaliya-Perumal et al., 2020). *Acvr1* mutation results in abnormally enhanced sensitivity of this receptor to BMPs, allowing for overexcitation of the BMP/SMAD pathway and heterotopic ossification. The typical feature of FOP is multiple skeletal deformities, involving fingers, toes, and cervical spine, and eventually resulting in pain, movement, and function limitation. POH is a genetic HO caused by inactivating mutations in the *GNAS1* gene, which result in decreased expression or function of the alpha subunit of the stimulatory G protein (Gsα) of adenylyl cyclase (Zhang et al., 2018). POH is characterized by intramembranous and cutaneous ossification, and could occur on the ear or fingers as an atypical phenotype (Kaplan et al., 1994; Zhang et al., 2018).

However, HO may be alleviated by physical intervention for traumatic-induced patients such as immobilization or Long-term bedridden. Doctors routinely use immobilization for extremity trauma patients (Kunz et al., 2014). But the mechanism that how immobilization protects the injury site reduces pain and improves healing remains unknown (Huber et al., 2020). Conversely, heterotopic ossification may become more severe in patients with insufficient immobilization and bed rest after fracture injury or joint surgery.

### Clinical Risk Factors

#### Physical Factors

There is a positive correlation between the formation of heterotopic ossification and force application. People who are over-exercised are more likely to develop heterotopic ossification (Jones et al., 2019). The explanation may be that more active people also have a higher probability of injury, excessive stretching of soft tissues leads to abnormal activation and differentiation of stem cells in local tissues, or that greater muscle mass leads to mechanical signal stimulation (Coons and Godleski, 2013; Dowdell et al., 2020; Rüdiger et al., 2020). Manifestations of heterotopic ossification due to mechanical stimulation can also occur in the temporomandibular joint (TMJ). Disturbance of occlusal forces will lead to TMJ disorder, while chronic abnormal forces and malposition of the joint will lead to heterotopic ossification of the TMJ (Jensen et al., 2010). Mechanics-based two- and three-dimensional finite element analysis and clinical findings indicate that the occurrence of heterotopic ossification after cervical total intervertebral disc replacement is characterized by a strong correlation with regional stress. Compressive force induces HO on the uncovered vertebral endplates, while shear force causes HO in the anterior upper and lower parts of vertebrae (Ganbat et al., 2014; Ganbat et al., 2016).

It is also quite common to apply some physical interventions, such as immobilization, physical therapy, intermittent activity, or massage for convalescent patients. However, the effect of those physical interventions on HO remains controversial. The transitory periods of forcible passive movements on immobilized arthrosis could produce HO in the soft tissues around the arthrosis within two to 5 weeks (O’Connor, 1998; Michelsson and Rauschning, 1983). The bone volume of HO was positively correlated with the duration of chronic bed rest and the frequency of forcible movement. Interestingly, HO was not induced when the limbs were merely immobilized without forcible movement, or merely passively movement without immobilization (Ellerin et al., 1999). Some researchers found that immobilization totally inhibited the formation of HO (Huber et al., 2020). Some researchers reported that surgery combined with postoperative physical therapy and rehabilitation program was effective to treat patients with heterotopic ossification of the elbow (Salazar et al., 2014). The reasons for this variation may be due to differences in the specific method, time of implementation, and duration of immobilization or rehabilitation exercises, besides the differences in the patients themselves collected in those clinical studies. It takes approximately 5–6 weeks for CT-visible heterotopic ossification to develop at the injury site, and early rehabilitation activities performed at inappropriate time points or approaches that apply additional forces to the injury site will likely result in a higher incidence of HO.

#### Spinal Cord and Brain Injuries

Neurogenic HO usually occurs following central nerve injuries, such as spinal cord injuries and cerebral lesions, and the prevalence has been reported to range from 10 to 53% (Teasell et al., 2010; Ranganathan et al., 2015). However, the mechanism that how the nervous system regulates HO formation remains incompletely understood. It has been demonstrated that peripheral neurotransmitters influence osteoblast formation, and the cortical bone density can be modulated by mechanistic-neural pathways (Huang et al., 2019; Zhu et al., 2019). Central neural signaling could precisely modulate bone metabolism and homeostasis. Leptin, as well as neuropeptide Y and cannabinoids, play an important role in the neural regulation of bone (Idris et al., 2005; Yue et al., 2016). However, it is unclear whether neural regulation of osteogenesis and osteolysis occurs in the same way as heterotopic ossification. The current researches are primarily devoted to the findings that osteogenic precursor cells in heterotopic ossification originate from the endoneurium and are strongly associated with local neuroinflammation leading to the blood-nerve barrier (BNB) penetration (Lazard et al., 2015; Olmsted-Davis et al., 2017; Davis et al., 2018). In general, thoracic and cervical spine injury can lead to more severe heterotopic ossification, which usually develops caudally at the level of injury, most commonly in the hip joint (Brady et al., 2018). Unlike spinal cord injuries, brain injuries often cause generalized heterotopic ossification, including hip, knee, and elbow or shoulder joints (Garland, 1988).

#### Empyrosis

In the case of burn patients, in addition to the typical clinical phenomenon of thermal injury, the occurrence of heterotopic ossification is also frequently observed. Heterotopic ossification is highly probable when the burned area is more than 20% of the body surface area (Mujtaba et al., 2019). In addition to the burn-induced cascade reaction that promotes heterotopic ossification formation, the scar tissue that forms around the periarticular will also limit the range of motion of the joint, which in turn may simultaneously influence heterotopic ossification from a biomechanical approach. Theoretically, the inflammatory cascades due to burns promote heterotopic ossification; the limited fixation due to burning scars may inhibit heterotopic ossification, or the mechanical force from small movements pulls on a large area of tissue due to scars, thus promoting heterotopic ossification. Furthermore, limited joint motion due to scar tissue may also confuse the clinical diagnosis of heterotopic ossification, which could also lead to restricted joint motion. Distinguishing between the two commonly relies on radiographic studies (Suito et al., 2018; Chen et al., 2019).

#### Surgery

Surgery that irritates the joint and its surrounding soft tissues may lead to the occurrence of heterotopic ossification. Following hip arthroplasty, the rate of heterotopic ossification occurrence could approach approximately 40% (Ranganathan et al., 2015). Surgery on the other joints, such as the knee, elbow, and temporomandibular joint, may also result in heterotopic ossification of the soft tissues surrounding them (Meyers et al., 2019). Surgery, especially invasive surgery, can lead to local tissue damage and pathologies such as ischemia and inflammation, which are high-risk factors predisposing to the development of heterotopic ossification. Generally, minimally invasive surgery (MIS), including MIS anterolateral (MIS-AL) and minimally invasive direct anterior approach (AMIS), could reduce the risk of HO compared with the standard modified anterolateral (STD-Watson-Jones) approach (Hürlimann et al., 2017).

#### Fracture

Fractures are an important risk factor for heterotopic ossification. Fractures usually result from trauma, and surgery to treat fractures is in turn invasive trauma to local tissues. HO following orthopedic injury occurs most frequently after acetabular fractures and elbow fractures. Interestingly, injury severity score, sex, and fracture type do not affect this risk, but long-term mechanical ventilation is the specific risk of HO (Firoozabadi et al., 2014). This is perhaps because of the impact of mechanical ventilation itself on the patient, such as anoxia; or because mechanically ventilated patients are typically bedridden for long periods, which may influence the traditional regulation of bone metabolism and the formation of heterotopic ossification from the mechanism of mechanical signal stimulation.

### Management and Treatment

#### Physical Therapy

The effect of physical therapy on heterotopic ossification is controversial, but physical factors, including postoperative rehabilitation exercises, joint immobilization, and prolonged bed rest, indeed influence heterotopic ossification. It has been shown that complete joint fixation can eliminate heterotopic ossification at the Achilles tendon in the mouse model (Huber et al., 2020). Others, however, believe that early postoperative exercise facilitates recovery and prevents the development of heterotopic ossification (Aronen et al., 2006; Ranganathan et al., 2015; Meyers et al., 2019). Physical therapy and continuous passive motion machines have been used for the postoperative management of total knee arthroplasty, for which a commonly encountered surgical complication is heterotopic ossification. Physical therapy has been found to be moderately beneficial at 3 months after total knee arthroplasty (Lowe et al., 2007; Manrique et al., 2015). A randomized controlled trial also found that physical therapy was superior for total hip replacement management (Mikkelsen et al., 2014). However, burn surgeons often find an increased incidence of HO in patients who are subjected to overly passive range of motion exercises at the elbow to prevent skin contracture (Meyers et al., 2019). The key to the discrepancy may lie in the duration and timing of the immobilization. In the early post-traumatic phase, immobilization facilitates the normal recovery of local tissues, while repetitive passive movements may lead to an aggravation of local micro-injuries, which in turn may lead to organization and ossification of soft tissues. However, in the late stage of trauma, the local micro-injury and inflammatory environment have been almost recovered, at this time the appropriate passive movement is conducive to the local tissue blood supply and physiological metabolic activities, and is beneficial to the normal recovery of soft tissues. On the contrary, long-term bed rest or immobilization may lead to the deterioration of local microcirculation status, and the abnormal local microenvironment may induce the aberrant differentiation of soft tissue stem cells into bone tissue, resulting in the occurrence of heterotopic ossification.

#### Pharmaceutical Prophylaxis

The development of traumatic heterotopic ossification, as previously mentioned, is in part secondary to surgery. It is necessary to take some appropriate clinical interventions to reduce the risk of postoperative heterotopic ossification. Currently, the preventive medications that are more routinely used for HO in clinical practice are NSAIDs and Bisphosphonates (Ranganathan et al., 2015; Meyers et al., 2019). Essentially, the origin of heterotopic ossification is the abnormal osteogenic differentiation of stem cells in soft tissues. NSAIDs could prevent heterotopic ossification by inhibiting the osteogenic differentiation of progenitor cells (Chang et al., 2007; Chang et al., 2009). However, the negative impact of NSAIDs on fracture healing while preventing heterotopic ossification has to be taken into account. Indomethacin increases the potential risk of long-bone nonunion after orthopedic injuries (Marquez-Lara et al., 2016; Duchman et al., 2019). Balancing the risk of heterotopic ossification with malunion fractures is the key to appropriate NSAID delivery.

Bisphosphonates are generally considered to be antiresorptive agents that induce osteoclast apoptosis and inhibit calcification. Yet some studies have indicated that it may have some preventive effect on heterotopic ossification, although this conclusion is still controversial (Vasileiadis et al., 2010; Zaman, 2012). Aside from the first generation, subsequent bisphosphonates generally only affect osteoclasts and thus are less likely to be able to inhibit the production of heterotopic ossification. However, bisphosphonates have indeed been found to be specifically effective in patients with burns and spinal cord injuries (Teasell et al., 2010; Ranganathan et al., 2015). This may be due to the anti-angiogenic effect of bisphosphonates, which reduces the occurrence of HO by depleting angiogenesis, or because the binding of bisphosphonates to calcium affects the mineralization of the bone matrix.

Some recent studies have also found that non-coding RNAs may have a therapeutic effect on heterotopic ossification, although the effect has yet to be demonstrated in large-scale clinical trials. MicroRNAs targeting DKK1 and vascular endothelial growth factor (VEGF), such as miR-17-5p, can alleviate the heterotopic ossification present in Ankylosing spondylitis (Qin et al., 2019). Similarly, microRNAs that can regulate osteogenic genes, such as miR-203, which targets RUNX2, can also inhibit heterotopic ossification (Tu et al., 2016). Further studies of these non-coding RNAs could contribute to the development of medicines that work precisely at the post-transcriptional level for the treatment of heterotopic ossification.

#### Radiation

Radiation therapy can be effective in preventing heterotopic ossification after hip arthroplasty. The incidence of heterotopic ossification without radiation after hip arthroplasty is up to 90%, while the rate decreases to about 25% after radiation therapy (Popovic et al., 2014). Appropriate prophylactic doses generally range from 400 to 800 cGy and are given 24 h before or 72 h after surgery, and 700 cGy (25%) administered postoperatively was more effective in preventing HO than 400 cGy (42%) (Popovic et al., 2014; Liu et al., 2017). Higher doses do not demonstrate increased prophylactic benefit, and may bring additional side effects, including progressive soft tissue contracture, delayed wound healing, non-union fracture, joint stiffness, potential oncogenesis, or inhibition of growth of hip implants (Hamid et al., 2010; Milakovic et al., 2015). However, the efficacy of radiation prevention in joints other than the hip has not been adequately studied.

#### Surgery

For heterotopic ossification antecedent to Booker IV Classification, complete surgical resection is achievable as the aberrant bone is free-standing with the hard bone tissue at the joint. Surgical removal is the ultimate treatment for patients who have limited effectiveness with other treatments and are unable to be completely cured (Łęgosz et al., 2019). However, it should be considered that surgical resection itself is an invasive stimulus, which may lead to the recurrence of heterotopic ossification after surgery, especially in susceptible subjects. Otherwise, despite the successful removal of the heterotopic ossified tissue, there is still a risk of recurrence after the surgery.

## Biological Mechanisms of HO

The type of ossification that occurs in heterotopic ossification differs depending on the origin of the HO. Among the hereditary HO, Progressive Osseous Heteroplasia (POH) and Albright hereditary osteodystrophy (AHO) are considered to be intramembranous ossification, while fibrodysplasia ossificans progressiva (FOP) is considered to be endochondral ossification (Kaplan and Shore, 2000). This is due to their different pathogenesis. In trauma-induced HO, it is generally accepted that this process occurs through endochondral osteogenesis (Wong et al., 2020). Although the precise mechanism has not been fully investigated, pathological staining such as SOFG on traumatic HO shows that cartilage formation occurs first and then ossification is formed based on it (Yu et al., 2021). However, it is worth exploring whether there is direct differentiation of MSC into osteogenic progenitor cells resulting in intramembranous ossification in traumatic HO. The single-cell sequencing results from the traumatic HO injury site showed that some of the MSCs differentiated into osteoblasts rather than chondrogenic cells (Huber et al., 2020). Moreover, this injury is usually accompanied by nerve and vascular damage. This osteogenesis of neuro- and vascular-derived cells may also affect the frequency of intramembranous vs. endochondral ossification (Wong et al., 2020).

### Cell Precursors of HO

One of the most significant differences between pathological heterotopic ossification and physiological osteogenesis is the distinct cellular source. The cellular origin of physiological osteogenesis is the differentiation of preosteoblast, but the precursor cellular origin of pathological heterotopic ossification has not been fully investigated. Table 1 summarizes the cell types that contribute to heterotopic ossification based on currently published studies. In general, the cellular origin of pathological osteogenesis is not limited to the osteoblast lineage, but potentially results from the pluripotent differentiation of a diverse range of stem cells.

**TABLE 1:** Cells types contributing to heterotopic ossification.

Study	Cell types	Findings
Feng et al. (2020)	Tendon-derived progenitor cells (Ctsk-Cre)	Ctsk could label progenitor cells of HO in tendon
Kan et al. (2018)	Interstitial/perivascular cells (Gli1-Cre)	Gli1-Cre lineage cells contribute to endochondral HO
Agarwal et al. (2017)	Tendon/periosteum/fascia (Scx-Cre)	Scx-cre lineage cells contribute to trauma-induced and BMP-induced HO
Olmsted-Davis et al. (2017)	Endoneurium (Wnt1-CreERT)	PS^+^ and SP7^+^ cells from peripheral nerves contribute to HO
Dey et al. (2016)	Endothelial/bone marrow/muscle interstitial cells (Mx1-Cre)	Mx1-Cre lineage cells contribute to intramuscular HO
Agarwal et al. (2015)	Mesenchymal progenitor cells (Nfatc1-Cre)	ca-ACVR1^fx/WT^/Nfatc1-Cre^+^ mice develop heterotopic ossification
Regard et al. (2013)	Mesenchymal progenitor cells (Prx1-Cre; Dermo1-Cre; Ap2-Cre)	Loss of Gnas mice resulted in PHO
Kan et al. (2013)	Pericyte/adipocyte/connective tissue interstitium (Glast-CreERT)	Glast-creERT labeled progenitors contribute to HO at all stages
Medici et al. (2010)	Endothelium/muscle satellite cells (Tie2-Cre/VE-Cadherin-Cre)	Endothelium/muscle satellite-derived cells contribute to HO

To be more specific, Ctsk was previously found to be able to label osteoclasts and periosteum stem cells. Recently, a subgroup of tendon-derived progenitor cells (TDPCs) was also found to be labeled by Ctsk (Feng et al., 2020). TDPCs, as stem cells in tendon tissue, are capable of multidirectional differentiation and would differentiate towards osteogenesis under certain conditions resulting in heterotopic ossification. In addition, mesenchymal stem cells in tendon areas could also be activated to osteogenic differentiation, which can be labeled by Nfatc1-Cre, Prx1-Cre, and Dermo1-Cre. It is possible that some other cells with proliferative capacity may also shift to osteogenic differentiation in some conditions. For example, perivascular cells (Gli1-Cre), PS^+^ and SP7^+^ cells from peripheral nerves, and muscle satellite cells (Tie2-Cre/VE-Cadherin-Cre) all contribute to HO. In conclusion, the cellular origin of HO is relatively complicated, and a variety of cells have the potential to shift to osteogenic differentiation in response to some specific stimulus, which in turn promotes HO formation.

### Inflammation and HO

Inflammation serves as an important microenvironmental alteration in the development of heterotopic ossification. Trauma leads to a state of local and systemic inflammation, resulting in elevated inflammatory cytokines, such as TNFα, IL-1β, IL-6, and MCP-1, which could cause abnormal activation of mesenchymal stem cells in the soft tissues (Sung Hsieh et al., 2017). Inflammation-associated cells, such as macrophages and mast cells, also accumulate at the site of trauma-induced heterotopic ossification and promote heterotopic ossification (Convente et al., 2018). Lymphoid tissues also contribute to the cellular niche in Heterotopic Ossification (Loder et al., 2016). The main role of inflammation is to turn MSCs, such as normal fibroblast lineage, into the osteogenic lineage, initiating the onset of heterotopic ossification.

### Hypoxia and HO

The hypoxic state of local tissues after trauma may also initiate heterotopic ossification. Regional tissue hypoxia causes the activation of Hypoxia-inducible factors (HIFs), consisting of 1 of 3 α subunits bound to HIFβ (Meyers et al., 2019). HIFs could increase the production of pro-angiogenic cytokines such as VEGF, facilitating localized pathological bone tissue formation (Dilling et al., 2010; Hwang et al., 2019). The inhibition of HIFs could attenuate HO formation in experimental models (Agarwal et al., 2016).

### Signaling Pathways and HO

Most of the fundamental research on heterotopic ossification is presently based on traumatic and genetic mouse models. In general, hyperactivation of bone morphogenetic protein (BMP) and consequent cascading activation of activin type-1 receptor (ACVR1) is thought to lead to abnormal endochondral osteogenesis, resulting in heterotopic ossification. The dysregulation of Hedgehog (Hh) signaling also contributes to many HO. However, recent studies have suggested that this pathological osteogenic process may share similar biological mechanisms with physiological osteogeneses, such as RUNX2, a classical osteogenic transcription factor (Kim et al., 2020). CK2/HAUSP pathway is a critical regulator of RUNX2 stability because Casein kinase 2 (CK2) phosphorylates RUNX2 and recruits the deubiquitinase herpesvirus-associated ubiquitin-specific protease (HAUSP) to stabilize RUNX2 away from ubiquitin-dependent proteasomal degradation. Meanwhile, regional osteoclast activities are also enhanced during the formation of heterotopic ossification, as the formation of the bone marrow cavities depends on a dynamic balance between osteogenesis and bone resorption. Furthermore, osteogenic-osteoclastic crosstalk, such as the transforming growth factor-beta (TGF-β) released after augmented osteoclastic activity that recruits mesenchymal stromal/progenitor cells (MSPCs) in the HO microenvironment for bone remodeling activities, also plays an important role in heterotopic ossification (Wang et al., 2018). PDGF-BB concentration was also increased during HO progression. Therefore, the bone formation process of heterotopic ossification is different but correlated to that of normal physiological osteogenesis.

Some proteins that affect bone morphology and bone development also influence the formation of heterotopic ossification. Bone morphogenetic proteins (BMPs) are required for multiple developmental processes, including bone and cartilage formation (Kaliya-Perumal et al., 2020). BMPs bind to ACVR1, which locates on the cell membrane surface phosphorylating SMAD1/5/9(8). Phosphorylated SMAD1/5/9(8) combine with SMAD4 and import into the nucleus, regulating transcription that drives endochondral ossification (Nosho et al., 2020). When BMP receptors bonded with Activin A, SMAD2/3 is activated to regulate inflammation (Rautela et al., 2019). The occurrence of FOP is also associated with the R206H mutant substitution of *Acvr1*, enhancing the response to various BMP ligands (Alessi Wolken et al., 2018). Retinoic acid receptors (RARs) are morphogens that impact both osteogenesis and chondrogenesis. There is a hypothesis that RAR agonism could impede HO formation by preventing the differentiation of prechondrogenic cells, and was partly tested in a subcutaneous rBMP2-induced HO model in mice (Cash et al., 1997; Shimono et al., 2010; Riedl et al., 2020). The Hedgehog (Hh) pathway also plays an important role in HO. Hh protein inhibits the GPCR-like protein Smoothened (SMO) by binding to the Patched (PTCH1) receptor, leading to SMO aggregation in cilia and phosphorylation of the cytoplasmic tail. SMO mediates downstream signaling and induces GLI protein detachment from SUFU. GLI1 and GLI2 proteins translocate to the nucleus to activate the transcription of Hh target genes (Regard et al., 2013; Feng et al., 2020). From this viewpoint, it can be inferred that biomolecules such as microRNAs, LncRNAs, and exosomes could also regulate heterotopic ossification by influencing some specific key proteins that regulate bone morphology and development, but this remains to further study.

## Mechanics and HO

### Mechanical Signals of HO

**FIGURE 1:**
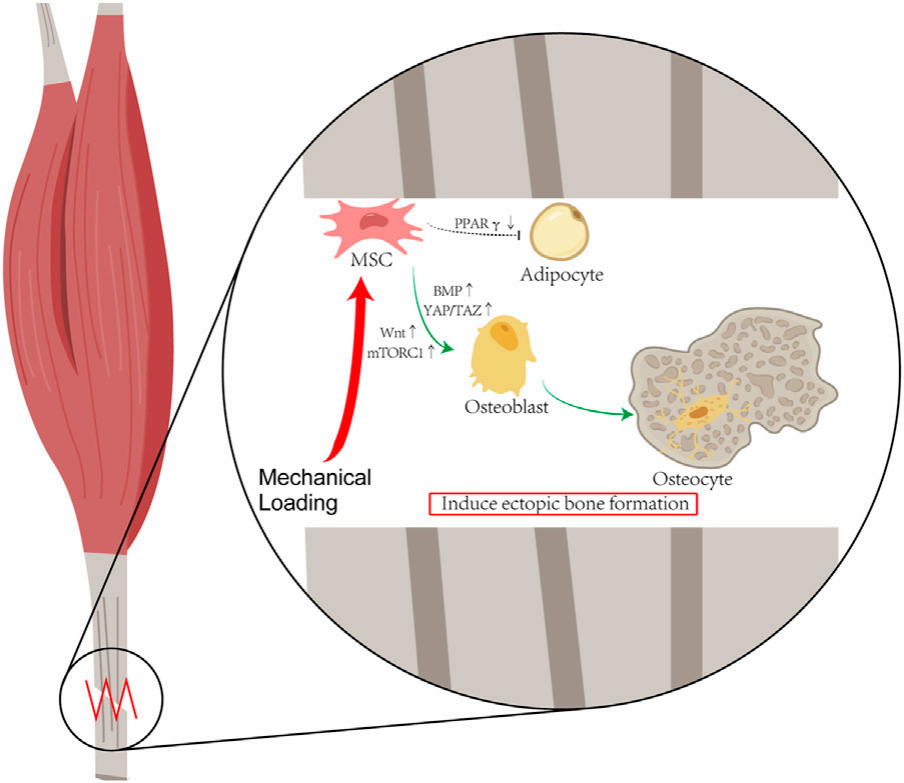
Hypothesis of Mechanical Stimulation of HO: Mechanical stress initiates osteogenic differentiation of mesenchymal stem cells (MSCs) in soft tissue. Stem cell fate of MSCs shifts from favoring lipogenic cells to osteogenic cells under mechanical loading. According to the published literature about HO, after the mechanical loading, the activations of the YAP/TAZ and mTORC1 pathway enable MSCs to differentiate into osteoblasts, and the decrease in PPARγ expression reduces the differentiation of MSC into adipocytes.

Heterotopic ossification can be modulated by mechanical signals. It is generally acknowledged that mechanical stress stimulation serves an important function in the physiological osteogenesis process. Osteocytes can sense local mechanical cues and thus induce bone formation, disuse-induced bone loss, and skeletal fragility (Qin et al., 2020). The primary mechanosensors in osteocytes include osteocyte cytoskeleton, dendritic processes, integrin-based focal adhesions, connexin-based intercellular junctions, primary cilium, ion channels, and extracellular matrix (Uda et al., 2017). It is now generally accepted that the traditional regulation of bone metabolism is deeply affected by mechanical stimulation signals. Current studies suggest that heterotopic ossification, a pathological osteogenic process, is modulated by mechanical signals as well. Mechanical stress initiates osteogenic differentiation of mesenchymal stem cells (MSCs) in soft tissue. Stem cell fate of MSCs shifts from favoring lipogenic cells to osteogenic cells under mechanical loading (Figure 1).

In the genetic-induced heterotopic ossification murine model, *Acvr1* mutant cells change the local microenvironment, resulting in the skewing of the threshold for mechanical stimuli and becoming more sensitive to the fate of chondral/osteogenic lineages (Haupt et al., 2019). Stanley’s study revealed that mechanistic signalings of *Acvr1* mutant cells in the soft matrix resemble that of non-mutant cells in the hard matrix, and are dependent on RhoA and YAP1 signaling (Stanley et al., 2019). Huber’s study found that mechanical stress can be transmitted to mechanosignaling receptors on heterotopic ossified mesenchymal progenitor cells through the extracellular matrix and cell adhesion, such as through focal adhesion kinase signaling and nuclear translocation of the transcription coenzyme TAZ, which regulates the progression of heterotopic ossification (Huber et al., 2020). However, the specific mechanism of Acvr1 in the mechanical signaling process is not clear, and no literature suggests a direct action in the mechanical signaling cascades. Because mutations in Acvr1 result in increased sensitivity to BMP, it is reasonable to believe that the Acvr1 response to mechanical stimulation is BMP-dependent.

Early studies have found that BMP-2, 4, 6, and 7 are differentially expressed depending on the mechanical stimulation (Rui et al., 2011). However, how BMPs can sense mechanical signals has been unclear for a long time, and only recently some studies have made advances. BMP-2 signaling senses mechanical signs because of the cross-talk with YAP/TAZ at the transcriptional level. In C2C12 cells, it was shown that Smad1/5/8 can be phosphorylated and translocated into the nucleus in the presence of BMP-2 signaling alone. However, activation of osteogenic genes requires cytoskeletal tension-induced nuclear accumulation of YAP/TAZ. BMP-2 signaling responds to mechanical cues by sensing nucleocytoplasmic shuttling of YAP/TAZ (Wei et al., 2020).

YAP and TAZ (also known as WWTR1) are two protooncogene proteins that are widely known as mechanosensors and mechanotransducers in various cell types (Dupont et al., 2011). The link between YAP/TAZ and mechanical signals is extensively explored in physiological osteogenesis as well as in osteogenic lineage. YAP/TAZ translocates from the cytoplasm to the nucleus depending on ECM stiffness in MSCs (Panciera et al., 2017), and mechanical niches trigger YAP/TAZ translocation contributing to osteoblastogenesis (Xiong et al., 2018). MST1/2 complexes with the scaffolding protein MOB kinase activator 1 (MOB1) to phosphorylate many proteins involved in chromatin condensation, apoptosis, and proliferation regulation, including cytoplasmic large tumor suppressor kinases 1 and 2 (LATS1 and LATS2). Activated LATS1/2, in turn, binds to YAP/TAZ and phosphorylates its serine, resulting in its retention in the cytoplasm and non-entry into the nucleus for function (Kovar et al., 2020). This part of YAP/TAZ pathway can interact with multiple signaling pathways at different levels, such as Hippo. In the process of heterotopic ossification, mesenchymal stem cells in soft tissues could be activated for osteogenic differentiation and become osteoblast rather than fibroblast after mechanical stimulation by YAP/TAZ conduction. Moreover, once MSC pluripotent differentiation leads to the initiation of the osteogenic procedure, mechanical stimulation further promotes the proliferation and differentiation of osteoblasts, resulting in enhanced heterotopic ossification (Yu et al., 2018). Simultaneously, osteoclast, as well as bone resorption activity, can also be affected by mechanical stresses, and even osteoclast-osteoblast crosstalk based on PIEZO1 could occur in response to mechanical forces (Wang et al., 2020). However, it is still unclear whether these osteoclast and osteoblast characteristics of normal bone tissue are completely identical in heterotopic ossification.

LRP5/6 is a key receptor in the Wnt signaling pathway. Wnt signaling plays a central role in the mechanotransduction of bone. But the mechanisms by which wnt signaling senses mechanotransduction signals specifically may be multi-pathway and multi-level. YAP/TAZ is still an important part of the Wnt pathway to sense mechanical signals. At the cell membrane, YAP/TAZ binds to Axin on LRP6, allowing the recruitment of β-transducin repeatase containing E3 ubiquitin-protein ligase (BTRC) to the β-catenin disruption complex (Azzolin et al., 2014). In the cytoplasm, YAP/TAZ binds to the cytoplasmic Wnt signaling transducer disheveled segment polarity protein 1 (DVL1) and inhibits its phosphorylation, thereby abrogating its translocation to the nucleus (Barry et al., 2013). Serine phosphorylated YAP and TAZ can also bind directly to β-catenin (Zhou et al., 2017). In addition, it can also function as a transcriptional co-activator. How the Wnt pathway specifically senses mechanical signals in bone metabolism has not been completely understood, but there is no doubt that the wnt pathway plays an important role in the biomechanics of bone.

In addition, mTORC1 signaling pathway serves as a mechanosensor modulating HO. Rodgers found that mTORC1 could activate quiescent stem cells into an “alert state” thus responding quickly to injury and stress conditions (Rodgers et al., 2014). The activation of mTORC1 promotes chondrogenesis and osteogenesis. Several studies have demonstrated that mechanical loading could activate the mTORC1 signaling pathway via inducing the phosphorylation of p70 S6 kinase (Lin and Liu, 2019). Chen found mechanical loading modulated HO of the tendon through the mTORC1 signaling pathway, furthermore, low elongation mechanical loading attenuated HO, while high elongation mechanical loading accelerated HO *in vivo* (Chen et al., 2017). Stimulated by mechanistic signaling, mTORC1 activates Sirtuin 1 (Sirt1) in the nucleus. Sirt1 is a histone deacetylase that acts as a novel bone regulator and represses the expression of sclerostin gene SOST, which is usually regarded as a strong negative regulator of osteoblast differentiation and bone formation (Liu et al., 2019). SOST inhibits β-catenin and osteogenic gene expression after binding to LRP5/6. Therefore, rapamycin, a selective mTORC1 signaling pathway inhibitor, is a potential therapeutic agent for heterotopic ossification.

### Mechanics and Stem Cell Fate

Mechanical interventions may affect HO formation by altering stem cell fate. Stem cells are able to sense their mechanical environments through various mechanosensors, including the cytoskeleton, focal adhesions, and primary cilia (Chen and Jacobs, 2013). The cytoskeletal tension could be generated by the interacts between myosin and actin, which is important for mechanically induced osteogenesis of stem cells. Focal adhesion is formed by the adapter proteins linking the cytoskeleton to integrins. Forces are transmitted based on these intact focal adhesions (Nardone et al., 2017). The primary cilium is a single, non-motile, antenna-like transmembrane structure, acting as a microdomain to promote biochemical signaling (Pala et al., 2017). Joint immobilization could reduce mechanotransduction signaling (Kunz et al., 2014). In the immobilized murine model, the fate of mesenchymal progenitor cells was altered. Mobile MPCs expressed more genes related to osteogenesis and chondrogenesis, such as *Sox9*, *Runx2*, *Spp1*, and differentiated more into osteogenic cells; immobile MPCs expressed more genes related to lipogenesis, e.g. *Fabp4*, *Pltp*, *Lrp1*, and differentiated more into lipogenic cells (Huber et al., 2020). In the osteogenic-lipogenic fate shifting of MSCs caused by mechanical stimulation, sclerostin signaling potentially serves as a significant regulator. Unloading makes the expression of the sclerostin increase, which downregulates two key osteogenic procedures: Wnt/β‐catenin signaling and YAP/TAZ transcriptional activity. The crosstalks between Wnt/β‐catenin and PPARγ influence the physiological balance between osteogenesis and adipogenesis (Benayahu et al., 2019). As the MSCs are mechanically stimulated and favor osteogenic differentiation, heterotopic ossification becomes severe. Conversely, when they favor lipogenic differentiation, the amount of heterotopic ossified bone decreases. Therefore, joint immobility after injury promotes adipogenesis rather than osteogenesis, leading to reduced HO formation. And the use of pharmacologic inhibitors altering mechanical signaling may prove to be an effective therapy that spontaneously induces adipogenesis at sites prone to osteogenesis. The accumulation of fatty tissue in the joint near the site of injury is much less severe than HO, leading to a more favorable outcome (McTighe and Chernev, 2014).

**FIGURE 2:**
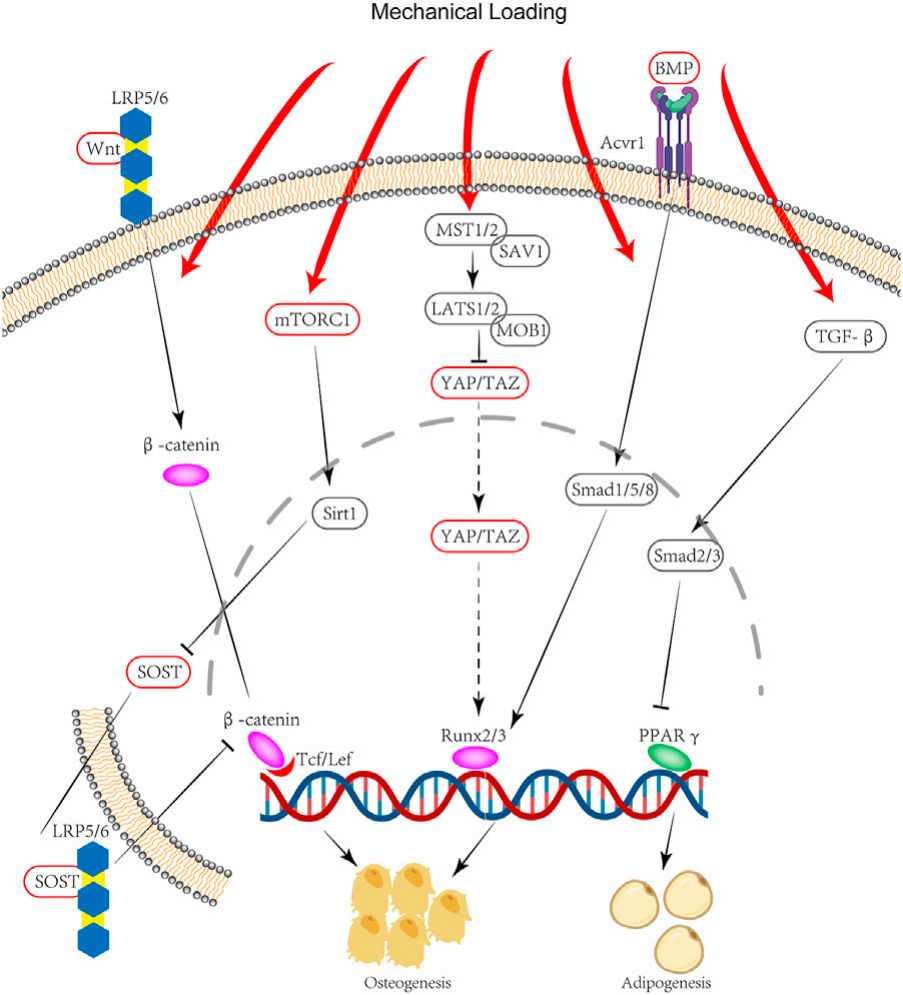
Signaling pathway of HO due to mechanical stimulation: Mechanical stimulation through mTORC1 leads to an increase in Sirt1 translocation into the nucleus, followed by a decrease in SOST secretion. SOST can bind to LRP5/6 to inhibit β-catenin. Mechanical loading can also activate Runx2/3 gene expression through YAP/TAZ. Thus mechanical stimulation promotes osteogenic gene expression through mTORC1 and YAP/TAZ. Meanwhile, mechanical stimulation can inhibit PPARγ gene expression through the TGF-β pathway, thereby suppressing lipogenic differentiation. These combined effects lead to a stem cell fate shift.

Mechanical loading has also been demonstrated to cause stem cell fate shift at the cellular level (Figure 2). Mechanical loading appears to favor osteogenesis whereas unloading conditions seem to promote adipogenesis. Passive stimuli including stiffness and viscoelasticity, as well as active stimuli including tensile/compressive stress and fluid shear stress, can affect cells through the extracellular matrix (Benayahu et al., 2019). Mechanical signals are conducted from the extracellular matrix through the cytoskeleton to regulate intracellular actions. Some important signaling pathways interact with mechanistic signals. For example, Wnt ligand binding to low‐density lipoprotein receptor‐related protein 5/6 (LRP5/6) coreceptors results in the translocation of β‐catenin to the nucleus and the enhanced transcription of genes that govern osteogenesis, and its interaction with the Hippo pathway that governs the activity of YAP/TAZ, which is regarded as an important mechanistic signaling transcription factor (Benayahu et al., 2019; García de Herreros and Duñach, 2019). Even cells that already have terminally differentiated into the myogenic lineage may be reconverted to the osteoblast lineage under certain conditions: C2C12, a myoblast cell line, can be converted to osteoblasts under the combined effect of BMP and mechanical stimulation (Wei et al., 2020). Although these studies demonstrate that cells of other lineages are capable of osteogenic differentiation, it is not clear whether the same phenotype occurs *in vivo*, resulting in heterotopic ossification.

Beyond biological experiments, a significant influence of local loading on the formation of heterotopic ossification has been found through the mechanobiological algorithm system. By designing a computational model of physiology that takes into account both mechanical and biological factors, Rosenberg found that modifications to the mechanical environment significantly alter the shape and production of heterotopic bone. Adjustment of load orientation, skin material characteristics, and location of maximum trauma resulted in four characteristic HO types. Simulation of negative pressure dressings and tourniquet application also served to highlight the behavioral characteristics of HO (Rosenberg and Bull, 2018). Still, the mechanobiological algorithm system needs further development to make it more compatible with the real world.

These basic studies suggest that mechanical signals contribute to the formation and development of heterotopic ossification, not only initiate heterotopic ossification through the activation of pluripotent differentiation of MSCs, but also influence the osteogenic program during HO by affecting osteocytes, osteoblasts, and osteoclasts. However, there are only a few studies related to mechanical stimulation and heterotopic ossification. Representative basic studies have only applied fixed models for attenuated mechanical stimulation, but elaborate force-added models also need to be investigated. Relevant clinical studies are even more lacking. Further studies in this direction would have guiding values for the development of new drug targets for the treatment of HO, as well as for the development of more effective clinical methods of physical therapy and prophylaxis for HO.

In summary, the effects of mechanics on heterotopic ossification could be considered from early, middle, and late stages, respectively. In the early stage of HO, mechanical stimulation may activate pluripotent differentiation of MSCs in soft tissues, e.g., mTORC1 could activate quiescent stem cells into an “alert state”, and promote chondrogenesis and osteogenesis, leading to HO initiation. Mechanical stimulation can alter stem cell fate, causing chromatin regions around osteogenic genes to open. This results in more expression of osteogenic-related proteins and promotes stem cell differentiation toward osteogenesis. Clinically, early post-trauma immobilization can attenuate or even prevent heterotopic ossification. In the middle stage of HO, which means heterotopic ossification has been triggered and pathologic ossification is in the process of formation. Since physiological osteogenesis and pathological osteogenesis have some commonalities, they both require stem cells to differentiate into osteoblasts, and the eventual ossifications are dependent on the function of osteoblasts performing osteogenic functions. Many fundamental signaling pathway, such as CK2/HAUSP/RUNX2 are necessary for both physiologic bone formation and HO. It can be assumed that the effects of mechanics on HO may be similar to that on the osteogenesis process. From the clinical perspective, patients at this stage may still need as much bed rest as possible to avoid stress on the trauma site and to prevent pathological osteogenesis. Conversely, for the late stage, prolonged immobilization may instead lead to local tissue inflammation and hypoxia, both of which are risk factors for heterotopic ossification, and may lead to tissue ischemia and necrosis along with malfunctioning. Therefore, for patients potentially suffering from heterotopic ossification in the initial stages of injury, early and adequate immobilization is essential to avoid stress on the injured area. For those patients who have been adequately immobilized after trauma, appropriate rehabilitation exercises are recommended in the late stages to prevent heterotopic ossification as well as promote functional recovery.

## Summary

HO is a diverse pathologic process. We still do not fully understand the cellular origin, pathogenesis, and underlying mechanisms of HO, and have not yet developed a specific treatment for HO beyond surgical resection. HO as a pathological osteogenic activity involving pluripotent differentiation of stem cells has many remaining aspects to be explored, although it has similarities to physiological osteogenic activity in some ways. This paper reviews the features of heterotopic ossification according to the established literature, with particular emphasis on the effect of mechanical stimuli on HO. However, the specific biological mechanism of this effect needs to be further investigated.

## Abbreviations

AHO, Albright’s hereditary osteodystrophy; BMP, bone morphogenetic protein; BNB, blood-nerve barrier; FOP, fibrodysplasia ossificans progressive; HIFs, Hypoxia-inducible factors; HO, Heterotopic ossification; MSPCs, mesenchymal stromal/progenitor cells; NSAIDs, Nonsteroidal Antiinflammatory Drugs; POH, progressive osseous heteroplasia; TDPCs, tendon-derived progenitor cells; TMJA, temporomandibular joint ankylosis; TMJ, temporomandibular joint.
